# Reaching vulnerable populations: lessons from the Global Fund to Fight AIDS, Tuberculosis and Malaria

**DOI:** 10.2471/BLT.16.179192

**Published:** 2017-02-01

**Authors:** Matthew Greenall, Osamu Kunii, Kate Thomson, Rene Bangert, Olivia Nathan

**Affiliations:** a The Global Fund to Fight AIDS, Tuberculosis and Malaria, Chemin de Blandonnet, 1214 Vernier, Switzerland.

Since its inception in 2002 – in partnership with civil society, donors, governments, people affected by the target diseases and the private sector – the Global Fund to Fight AIDS, Tuberculosis and Malaria has supported and championed country ownership of programmes that, together, have saved over 20 million lives.^1^

While substantial progress has been made, attempts to achieve target 3.3 of the sustainable development goals – i.e. to end epidemics of acquired immunodeficiency syndrome (AIDS), malaria, neglected tropical diseases and tuberculosis by 2030 – are being hampered because services are failing to reach vulnerable and excluded populations. This failure reflects the many deep-seated gender-related, human-rights-related, cultural, financial, political and social barriers that such populations face. Approaches that address the differences between diseases and between affected communities – while strengthening health systems – need to be developed, especially as countries become less reliant on donor funds.

The Global Fund has a responsibility to use its resources to achieve the greatest possible impact while ensuring the rights of those it serves are respected and promoted. This paper discusses how, in order to achieve these goals, the Global Fund has changed its approaches since 2002 and developed a new strategy for 2017–2022.

## Defining vulnerability

Decades of efforts to fight AIDS, malaria and tuberculosis have shown that a business-as-usual approach leaves many of the most vulnerable people behind. The Global Fund has always emphasized the need to reach key and vulnerable populations but initially provided little guidance on how to define and identify them. Country-level efforts supported by the Global Fund between 2002 and 2012 were often directed towards general population groups and, perhaps, failed to focus adequately on those who were most vulnerable.^2^ From 2013, the Global Fund began to develop context-based definitions that enabled countries to identify key and vulnerable populations for each targeted disease and for each epidemiological context (Box 1).

Box 1 **Definitions of key and vulnerable populations**

In 2016, the Global Fund to Fight AIDS, Tuberculosis and Malaria^3^ defined key and vulnerable populations as follows:

**Key populations:**

“Epidemiologically, the group faces increased risk, vulnerability and/or burden to at least one of the three diseases – due to biological, socioeconomic and structural factors. Access to relevant services is significantly lower for the group than for the rest of the population. Dedicated efforts and strategic investments are required to expand coverage, equity and accessibility. The group faces frequent human rights violations, systematic disenfranchisement, social and economic marginalization and/or criminalization, increasing vulnerability and risk and reducing access to services.”

**Vulnerable populations:**

“People whose situations or contexts make them especially vulnerable, or who experience inequality, prejudice, marginalization and limits on their social, economic, cultural and other rights.”

## Encouraging investments

Since 2013, the Global Fund has also updated its policies and procedures to enhance the effectiveness and scale of programmes targeted at vulnerable groups. The Global Fund considers community voices to be a critical component of grant design. While the Global Fund has always required countries to establish country coordinating mechanisms that included community representation, the associated guidelines were revised in 2013 to ensure that women and key populations had a voice. Since then, any country funding requests have had to be based on a broad nationwide dialogue that included all vulnerable groups. The participation of key and vulnerable populations was bolstered by the Global Fund’s special initiative on community, human rights and gender. This provided communications outreach to community and civil society groups, dedicated technical support and long-term capacity building – with a particular focus on key and vulnerable populations. Simultaneously, funding application forms were revised to improve attention to key and vulnerable populations. For example, applicants are required to describe any known gaps in coverage and any gender-related and human-rights-related barriers that contribute to vulnerability. A modular framework, which applicants use to describe investments and anticipated results, provides a clear analysis of the levels of funding directed to targeted programmes for key populations and to programmes designed to remove human-rights-related barriers that systematically exclude certain groups. Applicants are asked to describe how they would address such barriers when delivering their proposed programmes. This process was further supported by the publication of guidance notes – which summarize the evidence for effective programming among excluded groups – and the launch of a special initiative on country data systems. The latter initiative was designed to help countries improve their estimates of the sizes of key populations and, particularly, groups that are criminalized or excluded – e.g. gender and sexual minorities in the context of human immunodeficiency virus (HIV).

As these new approaches were being developed, the Global Fund made available 200 million United States dollars (US$) for regional grants, to invest in populations not covered by country grants and to scale up relevant interventions – especially in environments where exclusion and vulnerability were being exacerbated. Some of the regional funding was used on miners affected by tuberculosis in southern Africa, migrant populations in the malaria-affected Greater Mekong subregion and key populations for HIV in several areas of the world. In addition, an emergency fund provided rapid funding to vulnerable populations – e.g. refugees and the displaced in conflict situations and other humanitarian emergencies.

## Promising signs of progress

While it is too soon to provide categorical evidence of success, the Global Fund appears to be moving in the right direction. Over the 2014–2016 funding cycle, the number of countries with key population representatives in their country coordinating mechanisms increased from 53 to 61, the proportion of all members of such mechanisms who are female increased from 34% to 40% and the number of countries with accurate population size estimates for female sex workers and men who have sex with men increased from 32 to 55.^4^

In its report on the 2014–2016 funding cycle, the Global Fund’s technical review panel stated that “the focus of proposal requirement and the emphasis on impact have encouraged increasing attention to programmes for key and vulnerable populations”.^5^ Notable examples of this increasing attention include the decisions, by Botswana and the Democratic Republic of the Congo, to allocate Global Fund financing to programmes for key populations for the first time. There has been a substantial increase in funding for harm-reduction programmes in African countries and the quality of such programmes, which increasingly include comprehensive rather than selective packages, appears to be improving.^5^

There has been similarly increasing support for gender-responsive programming and initiatives aimed at girls and women – e.g. cash-transfer programmes for 30 000 vulnerable girls and social impact bonds for over 24 000 female sex workers in South Africa and investments of over US$ 50 million on programmes against gender-based violence across 30 countries.^4^ The proportion of the Global Fund’s investments focused on girls and women increased from 46% in 2010 to 60% in 2015.^1^

## Building for the future

While there are encouraging indications of progress between 2013 and 2016, the Global Fund can –and must – do much more. The Technical Review Panel’s report^5^ cited above noted that, while most included stronger analysis of vulnerable and key populations, many grant applications – and especially those for programmes against malaria and tuberculosis – did not include realistic or sufficiently large-scale plans to reach such groups.^5^ By 2016, some countries were entirely dependent on the Global Fund and other external funders for programmes with key populations and this raises concerns about the programmes’ financial and political sustainability.^5^

The Global Fund’s 2017–2022 strategy strengthens efforts to address the challenges posed by poor access to key and vulnerable populations in at least nine ways: (i) by the continuation and strengthening of support for engagement of vulnerable communities in grants and for collection of robust data on key and vulnerable populations – e.g. through a renewed strategic initiative on community, gender and human rights; (ii) by the development of in-house capacity – e.g. staff training on key and vulnerable populations and the establishment of advisory and working groups on related issues; (iii) by the revision of funding guidelines to reflect emerging evidence and good practice on programming for key and vulnerable populations; (iv) by the introduction of so-called matching funds, whereby selected countries deciding to use substantial portions of their funding for programmes related to key and vulnerable populations will be able to access additional funds to bolster these efforts; (v) by increasing focus on sustainability and transition from Global Fund support – e.g. transition planning and requirements for countries to allocate domestic funding to key and vulnerable population programmes; (vi) by promoting a shift from disease-specific to integrated service-delivery platforms – e.g. comprehensive antenatal care services that include the control of malaria in pregnancy, the prevention of mother-to-child transmission of HIV and tuberculosis screening; (vii) by scaling up mechanisms to improve feedback from service users – e.g. through funding key-population-led treatment and human rights observatories; (viii) by adapting systems to enable grant implementers to channel funding to small, unregistered community groups that are well placed to deliver services to those not accessing health facilities; and (ix) by the development of a stronger accountability framework to enable the Global Fund to improve its understanding of how it is performing with key and vulnerable populations and make adjustments as needed.^6^ By strengthening community responses and systems, one objective of the new strategy – the building of resilient and sustainable systems for health – should improve access for excluded groups. New key performance indicators focus on the coverage of key populations with comprehensive programming, the reduction of HIV incidence among adolescent girls and young women aged 15–24 years and the scale-up of comprehensive programmes to address human-rights-related barriers to access.^7^

If the most vulnerable and excluded groups are to be provided with health care in a sustainable, effective way, those groups must be able to inform, reshape and strengthen resilient and sustainable systems for health. As this brief paper shows, the Global Fund continues to improve and adapt its attempts to achieve these goals.
